# Cardiovascular–Kidney–Metabolic (CKM) Syndrome Staging and Relevance to Precision Nutrition

**DOI:** 10.3390/nu18091430

**Published:** 2026-04-30

**Authors:** Ghada A. Soliman

**Affiliations:** 1 Graduate School of Public Health and Health Policy, City University of New York, New York, NY 10027, USA; ghada.soliman@sph.cuny.edu; Tel.: +1-646-364-9515; 2 Advanced Science Research Center (ASRC), Structural Biology Initiative, City University of New York, New York, NY 10031, USA

**Keywords:** cardiovascular–kidney–metabolic syndrome (CKM), cardiometabolic risk, metabolic dysfunction-associated liver disease, nutrient-sensing, insulin resistance

## Abstract

**Background/Objectives:** It is estimated that one in three adults in the US has one or more risk factors for cardiovascular–kidney–metabolic (CKM) syndrome. The American Heart Association (AHA) has warned that the interaction between obesity, Type 2 diabetes (T2D), chronic kidney disease (CKD), and cardiovascular disease (CVD) leads to a multistage CKM syndrome with elevated mortality. This narrative review describes the newly coined terms CKM health and CKM syndrome, introduced by the AHA Presidential Advisory in 2023. **Methods**: In this narrative review, we will discuss the epidemiology and development of CKM syndrome, CKM stages, and the possible impact of precision nutrition on CKM and evaluate what is currently known about the role of nutrient metabolism in the physiological state and pathogenesis of CKM. **Results**: Since the AHA defined CKM syndrome in 2023, several studies have analyzed NHANES data to identify the correlations between CKM stages and adverse health outcomes. Studies also found that correlations between dietary intake and diet patterns may contribute to the protection against progression through various stages of CKM. However, experimental research and clinical studies are still lagging. Although the liver plays an integral role in nutrient metabolism, energy homeostasis, protein synthesis, nutrient storage, antibody production, and detoxifying compounds, it has not been included in the definition of CKM. **Conclusions**: Integrated body systems contribute to the development and progression of CKM. Precision nutrition and dietary patterns may play a role in the management of CKM and related comorbidities. Further research is warranted to address the role of precision nutrition in the prevention, early detection, and intervention in CKM syndrome as part of a comprehensive approach. It would be worth considering including metabolic dysfunction-associated liver disease (MASLD) and metabolic dysfunction-associated steatohepatitis (MASH) within the CKM framework.

## 1. Introduction

Cardiovascular Disease (CVD) is a leading cause of death in the US and is associated with multiple comorbidities, including diabetes mellitus and arterial hypertension [1]. In 2001, the American Heart Association (AHA) and American Diabetes Association (ADA) coined the term cardiometabolic risk to describe a global risk that encompasses CVD, type 2 diabetes mellitus, and obesity, as these conditions share a cluster of risk factors both independently and collectively [2,3]. Recently, the AHA recognized a framework for staging cardiovascular–kidney–metabolic (CKM) health and CKM syndrome to raise awareness of the interrelatedness of these multisystem chronic diseases [4,5]. The burden of disease from the collective CVD, CKD, Type 2 Diabetes (T2D), metabolic syndrome, and metabolic dysfunctions is significant in the US [6]. CKM syndrome presentation ranges from a healthy individual (stage zero) to patients with CVD and CKD (stage four), as shown in Table 1. Watson et al. analyzed data from US adults (N = 2,673,529) from 2013 to 2023 odd years obtained from the Behavioral Risk Factor Surveillance System (BRFSS) [7]. The authors concluded that approximately 6 in 10 young adults, 8 in 10 midlife adults, and 9 in 10 older adults in the USA report one or more chronic conditions [7].

Methodology: PubMed and CINAHL databases were searched for the following keywords: cardiovascular–kidney–metabolic (CKM), cardiovascular–kidney–metabolic syndrome, and nutrition, and/or nutrients. The search years were 2020–2025; the PubMed/CINAHL search yielded 158 articles, published from 2023 (when the CKM term was introduced) through 2025. Inclusion criteria: All peer-reviewed articles written in English. Exclusion criteria are articles not written in English or that did not have the term CKM. This narrative review is intended to summarize what is currently known about CKM syndrome, which is largely limited to observational studies, electronic medical records, and animal models, and therefore encourages future experimental research, clinical studies, randomized controlled trials, mechanistic studies, and causal inference studies.

CKM syndrome is a multisystem disease that stems from dysfunction of the integrated physiological body systems that maintain metabolic homeostasis, energy production, nutrient utilization, urea excretion, and electrolyte balance. The recent staging of CKM syndrome, as defined by the AHA, indicates that dysregulation begins with adipocyte dysfunction or excess adipocytes and abdominal obesity (Stage 1), and may include impaired glucose tolerance and prediabetes. As a continuum, Stage 2 shows risk factors of metabolic syndrome, which then progress to moderate and elevated risk of CKD and preclinical CVD (Stage 3), and clinical CVD (Stage 4). Therefore, it is plausible that nutritional interventions and lifestyle changes could be effective strategies in the early stages of CKM. However, current research on CKM syndrome relies on observational studies, which can only show correlations; therefore, clinical research and randomized controlled trials are warranted to assess the impact of nutritional interventions and healthier lifestyles on the prevention and early detection of CKM. Taken together, the data provide a justified need for a comprehensive, evidence-based assessment to support prevention, risk reduction, early detection, and management of CKM.

## 2. Cardiovascular-Kidney-Metabolic Syndrome Definition

The AHA statement conceptualized the pathophysiological interrelationships among CVD, metabolic diseases, and CKD as CKM syndrome. This connection would have significant implications for the prevention, early detection, assessment, and therapeutic management of CKM [4,5,8]. The term CKM reflects the interplay of obesity, type 2 diabetes mellitus (T2D), metabolic syndrome, CKD, and CVD.

## 3. Pathophysiology and Stages of CKM

There remain significant gaps in our understanding of the pathophysiology and mechanistic pathways of CKM syndrome, which warrant further investigation to improve health outcomes. As mentioned earlier, CKM is usually initiated by excessive or dysfunctional adipose tissue, leading to increased proinflammatory adipokines and cytokines, such as retinol-binding protein 4 (RBP4), cytotoxic T cells and National Killer (NK) cells, and oxidative products [9]. These proinflammatory mediators reduce insulin sensitivity, leading to glucose intolerance, insulin resistance, prediabetes, and T2D. In addition, bioactive and dysfunctional adipocytes increase oxidative stress, damaging endothelial and arterial walls and contributing to atherosclerosis and CVD. These proinflammatory and prooxidative mediators could also lead to the development of metabolic dysfunction-associated steatotic liver disease (MASLD), which amplifies the inflammation and may lead to liver failure. In addition, the release of proinflammatory and prooxidative mediators in the systemic circulation leads to glomerulosclerosis and tubular inflammation, resulting in albuminuria and proteinuria and thereby exacerbating the metabolic risk. The interaction between the kidney and heart inflammation through direct and indirect metabolic pathways leads to the advancement of CKD and CVD, which could progress to a multi-organ failure [10].

According to the AHA presidential advisory [10], CKM arises from dysfunctional/excess adiposity and abdominal obesity; thus, body mass index (BMI) and waist circumference (WC) may be the initial indicators of CKM. While a BMI > 25 kg/m^2^ is commonly used to screen for obesity, it is not an optimal measure, as it overestimates the BMI in athletes and does not account for body composition or muscle-to-fat ratio. Thus, measuring the WC in conjunction with BMI is preferable and more generalizable. Additionally, it is recommended to use a lower threshold, e.g., BMI > 23 kg/m^2^ (WC >80 cm in women, 90 cm in men), for individuals of Asian ancestry, as they are predisposed to metabolic abnormalities even at lower adiposity levels.

The AHA Presidential Advisory recommended a staging framework to optimize care for patients with multiple comorbidities and coined the term CKM. Briefly, in Stage 0, patients have no risk factors for CKM, with normal BMI and WC, normal blood glucose, and a normal lipid profile (Table 1). In Stage 1, patients exhibit signs of dysfunctional/excessive adiposity, including a BMI >25 kg/m^2^ and abdominal obesity, as indicated by a WC >88 cm in women and >102 cm in men. They may have impaired glucose tolerance or prediabetes mellitus, and blood pressure may be normal or elevated. In Stage 2, individuals exhibit elevated metabolic risk, as evidenced by higher BMI, abdominal obesity, T2D or metabolic syndrome, abnormal lipid profile with hypertriglyceridemia, and a moderate-to-high risk of CKD, as evidenced by low eGFR or albuminuria that persists for more than 3 months. While CKD was traditionally diagnosed by low eGFR, it has been shown to be unreliable [5]. Therefore, albuminuria >30 mg/g creatinine in a spot urine specimen is a more reliable screening metric for patients with T2D and an independent predictor of future CVD events [4]. In Stage 3, all signs from Stage 2 are present, as well as CKD, with the addition of subclinical CVD, as manifested by increased coronary artery calcium score, elevated cardiac biomarkers, or according to the Kidney Disease Improving Health Outcome (KDIGO) guidance heatmap [11]. The progression from subclinical to clinical CVD is designated as Stage 4. In Stage 4, patients could have clinical CVD, such as ischemic heart disease, peripheral artery disease, cerebrovascular disease, arrhythmia, and/or heart failure (Table 1). The following paragraphs summarize what is currently known about the epidemiology of CKM, including incidence, prevalence, mortality, and the impact of socioeconomic disparities on disease progression.

**Table 1:** CKM stages as defined by the American Heart Association Presidential Advisory (2023).

	Risk	BMI	WC	Glucose	BP	Lipid Profile	CKD	Subclinical CVD	Clinical CVD
Stage 0	No risk factors	Normal	Normal	Normal	Normal	Normal	No	No	No
Stage 1	Dysfunctional adiposity	≥25 kg/m^2^	Abdominal obesity (WC > 88 cm in women, 102 cm in men)	Impaired glucose tolerance or prediabetes	Normal or high	Normal	No	No	No
Stage 2	Metabolic risk factors	≥25 kg/m^2^	Abdominal obesity (WC > 88 cm in women, 102 cm in men)	Diabetes or MetS	Hypertension	Hypertriglyceridemia ≥ 135 mg/dl	Moderate/high risk CDK (low eGFR or albuminuria > 3 months)	No	No
Stage 3	Subclinical CVD	≥25 kg/m^2^	Abdominal obesity (WC > 88 cm in women, 102 cm in men)	Diabetes or MetS	Hypertension	Hypertriglyceridemia ≥ 135 mg/dl	Moderate/high risk CDK (low eGFR or albuminuria > 3 months)	Yes. Increased coronary artery calcium score or elevated cardiac biomarkers	No
Stage 4	Clinical CVD	≥25 kg/m^2^	Abdominal obesity (WC >88 cm in women, 102 cm in men)	Diabetes or MetS	Hypertension	Hypertriglyceridemia ≥ 135 mg/dl	Moderate/high risk CDK (low eGFR or albuminuria > 3 months)	Yes. Increased coronary artery calcium score or elevated cardiac biomarkers	Yes, ischemic heart disease, cerebrovascular disease, peripheral artery disease, arrhythmia, or heart failure

Table 1 summarizes the Cardiovascular-Kidney-Metabolic CKM) Syndrome as adapted from the American Heart Association Presidential Advisory 2023 [5,10]. At Stage 0, there are no risk factors, followed by a multistage progression from Stage 1 to 4, as shown in Table 1. Abbreviations: body mass index (BMI) > 25 kg/m^2^, waist circumference (WC), metabolic syndrome (MetS), blood pressure (BP), chronic kidney disease (CKD), cardiovascular disease (CVD).

## 4. CKM Epidemiology

The purpose for including the epidemiology of CKM is to explain the burden of CKM syndrome, establish a pattern of the disease state, and discuss its relevance to population science and communities, thereby providing a rationale and justification for delving deeper into the intricacies of the CKM syndrome and the need to embark on personalized nutritional intervention strategies to combat early stages of the CKM syndrome. Such precision nutrition interventions may include management of overweight and abdominal obesity (Stage 1) and interventions for prediabetes, diabetes mellitus, and high blood pressure (Stage 2), which are modifiable risks that can help reduce the CKM syndrome burden.

### 4.1. CKM Prevalence

Recently, Aggarwal et al. conducted a cross-sectional study to assess the prevalence and progression of CKM syndrome over time using the National Health and Nutrition Examination Survey (NHANES) data on US adults from 2011 to 2020 and employing a multistage probability design [12]. Alarmingly, the study revealed that 25% of US adults meet the criteria for Stage 1 CKM, 49% for Stage 2, 5.4% for Stage 3, and 9.2% for Stage 4. Thus, collectively, approximately 89.5% of US adults fall within the CKM stages, with older adults likely to be at an advanced stage of the disease, and black men at increased risk, which suggests subpopulation differences and ethnic disparity [12]. Likewise, in a nationally representative, longitudinal population-based cohort in South Korea, Yim and colleagues reported a similar pattern, with 43% of adults meeting the CKM Stage 2 criteria. In comparison, people with Stage 0 are more prevalent in South Korea than in US adults (21% versus 10.6%) [13,14,15]. In a Chinese population-based and hospital-based study, data from three diverse cohorts showed findings similar to those from NHANES in the US, with advanced CKM stages in patients with T2D [16].

### 4.2. CKM Incidence

Caudel and colleagues analyzed the NHANES longitudinal data of participants from cohort 1999–2018 and found that the 15 year adjusted cumulative incidence of mortality with each CKM stage was Stage 0 (5.5%), Stage 1 (5.7%), Stage 2 (7.9%), Stage 3 (8.7%), and Stage 4 (15.2%) with gradual increase in cardiovascular mortality [17]. It is worth noting that observational studies are correlational, not causational and thus experimental studies and randomized controlled trials are needed to address causality.

### 4.3. CKM Mortality

Kim and colleagues analyzed data from 877,537 participants in the Korean National Health Insurance database (2009–2012) to identify those who had two health checkups during that period. They reported that 15.3% of participants showed CKM stage progression, and that was correlated to a higher risk of all-cause mortality, heart failure, stroke, and myocardial composite outcome [18]. Another study utilizing the same database, but including 1,497,913 participants who underwent two health checkups during the same timeframe, also found that stages of CKM were positively associated with all-cause mortality and cardiovascular adverse outcomes [19]. The authors advocated prioritizing the identification of individuals at higher risk for CKM for early detection and implementing targeted therapeutic management to improve health outcomes. Interestingly, a study of 1745 patients with acute heart failure in Switzerland and Kyrgyzstan showed that CKM comorbidities were associated with increased risk of adverse outcomes and increased all-cause mortality [20]. The authors recommended a comprehensive CKM assessment and management in patients with acute heart failure. In the US, CKM-attributable mortality data from 2010 to 2019 were obtained from the CDC Online Data for Epidemiologic Research [21]. The results showed that CKM-attributable mortality in the US from 2010 to 2019 was more than three million deaths (age-adjusted mortality rates: 12,554/100,000), with associated age, racial, and regional disparities [21].

Two groups of investigators used data from the NHANES cohort (1999–2018; n = 18,350 and n = 27,909, respectively) and reported that patients with advanced CKM stage had reduced life expectancy and increased all-cause mortality [22,23]. Racial and ethnic disparities and sex differences were also observed [22,23]. Further, among adults with Stage 3–4 CKM, increased all-cause premature death (death before 75 years) was associated with adverse social drivers of health conditions, such as unemployment, lack of insurance, low family income, or not living with a partner [23]. Similarly, Ashworth and colleagues conducted a longitudinal study in an urban multiethnic borough in the UK using data from 332,353 participants [24]. The results also showed that social deprivation and ethnicity were major independent determinants of comorbidity [24].

## 5. CKM and Nutrition

As mentioned earlier, CKM Stage 1 is characterized by excessive adiposity and abdominal obesity, with possible impaired blood glucose control and prediabetes (Table 1). Given that overnutrition, as evidenced by overweight and obesity, is a component of malnutrition, management of patients with obesity through dietary interventions, reducing intakes of discretionary calories and energy-dense nutrients while promoting healthier lifestyles, is an actionable strategy for prevention, early detection, and management of CKM syndrome. The next section summarizes what is currently known about CKM and energy-yielding nutrients, micronutrients, dietary patterns, and different diets. Currently, studies on CKM are primarily conducted in animal models and correlational observational studies; therefore, causal inference studies and clinical trials are warranted.

### 5.1. CKM and Protein Metabolism

Protein is one of the three energy-yielding macronutrients; the other two are carbohydrate and fat. However, proteins play an essential role in every biological process, as they uniquely contain an amino group (NH_2_), in addition to carbon, hydrogen, and oxygen found in carbohydrates and fats. Proteins and their building blocks, amino acids, serve as precursors for enzyme synthesis, hormone regulation, structural elements, transport and storage, muscle synthesis, and immune response. Since inflammation and oxidative stress contribute to disease progression, the Dietary Inflammatory Index (DII), composed of 45 individual nutrients or foods, is used to quantify the inflammatory potential of foods [25]. The commonly used DII biomarkers are C-reactive protein, interleukin 6 (IL- 6), IL-1β, IL-4, IL-10, and tumor necrosis factor alpha (TNF-α). A lower DII score reflects a favorable anti-inflammatory dietary pattern, including a plant-based protein [26]. On the other hand, a higher DII score reflects a pro-inflammatory diet, which usually includes animal-based proteins.

To mimic CKM pathophysiology, Carvalho and colleagues subjected male C57BL/6J black mice to an early-life unilateral nephrectomy to induce renal dysfunction and fed them a diet rich in sugar and fat for 12 weeks to emulate the Western diet and cause CVD, and high in salt to induce metabolic syndrome and arterial hypertension simultaneously [27]. For the control group, the authors used sham-operated mice fed a standard chow. In this animal model of CKM, researchers compared the intake of a dietary additive supplement, Flexovital (FLX), containing beetroot extracts and the amino acids L-arginine and L-citrulline. The mice showed increased BMI, adipocyte area, and fat-to-lean-muscle ratio, which were reduced by FLX intake, suggesting the protective effects of nitric oxide-enhancing food additives in animal models [27,28]. The putative protective mechanism of FLX enhances nitric oxide (NO) availability, thereby decreasing metabolic and oxidative stress. The beetroot ingredient in FLX may activate or increase the expression of nuclear factor erythroid 2-related factor 2 (Nrf2), which may lead to antioxidative, anti-inflammatory, and cytoprotective properties [29] and may improve the endothelial functions [27,28,30]. Additionally, studies in animal models have shown that supplementation with maternal glycine, citrulline, taurine, cysteine, tryptophan, and branched-chain amino acids may be associated with the prevention of CKM phenotypes in offspring [31,32,33,34].

### 5.2. CKM and Carbohydrate Metabolism

Carbohydrates are the main dietary fuel and are hydrolyzed to glucose for energy generation through glycolysis, followed by the tricarboxylic acid cycle (TCA) cycle and the electron transport chain. Insulin is required in the peripheral tissues, such as muscle and adipose tissue, to facilitate the internalization of glucose for cellular energy production. Reduced peripheral tissue responsiveness to insulin leads to insulin resistance (IR), elevated triglycerides, and T2D. Thus, glucose disposal rate and the glucose triglycerides index (TyG) are used as surrogates for IR, as they reflect elevated fasting blood glucose and triglyceride concentrations [35,36,37]. The TyG index measures the natural logarithm of fasting blood glucose multiplied by fasting triglycerides, both of which are elevated in insulin resistance.

In CKM, impaired glucose tolerance and prediabetes mellitus are shown in CKM Stage 1, and hypertriglyceridemia is observed in Stage 2 (Table 1). Wang and colleagues noted that participants with higher TyG and estimated glucose disposal rate (eGDR) had a higher stroke rate compared to those with low TyG and eGDR (HR = 2.84; 1.67–4.81 CI) [38]. Remarkably, data from 3687 participants with CKM in the China Health and Retirement Longitudinal Study (CHARLS) cohort, 2011–2020, revealed that the TyG index and body roundness index (BRI) could serve as biomarkers for deterioration from pre-frailty to frailty in patients with CKM. Thus, the TyG and BRI could be used as a guide for early intervention and management for better health outcomes [39].

### 5.3. CKM and Fat Metabolism

As mentioned above, elevated triglyceride levels are considered a progression to Stage 2 CKM [5]. Most triglycerides are transported on the VLDL particles and on chylomicrons after meals. Studies in animal models showed that maternal intake of saturated fatty acids led to the development of dyslipidemia, T2D, and fatty liver in the offspring [33,34] and in mouse models [40,41]. On the other hand, supplementation with fish oil, an omega-3 polyunsaturated fat, during pregnancy has been shown to reverse CKM in rat offspring, as evidenced by decreased CVD and arterial hypertension [42,43]. Although studies in rats and mice showed protective effects against obesity during gestation, a systematic review and meta-analysis of randomized controlled trials (RCTs) in humans did not find that supplementation with fish oil during pregnancy and lactation reduced the risk of obesity in children [44]. One likely explanation is that, unlike guinea pigs, rodents have lipid metabolism and lipoprotein processing that differ from those of humans. As rodents, rats and mice carry most cholesterol in the High Density Lipoprotein (HDL) fraction, not Low Density Lipoprotein (LDL), unlike humans [45]. Also, they lack a functional cholesteryl ester transfer protein (CETP), which transfers cholesterol between lipoproteins. Therefore, the lipid and lipoprotein metabolism in rodents differs from that in humans [46]. Another alternative explanation is that the RCTs had small sample sizes and were heterogeneous [44].

### 5.4. CKM and Vitamins

Most water-soluble B vitamins can be classified by their roles in energy metabolism and homeostasis. In contrast, water-soluble vitamin C has multiple functions, including collagen synthesis, tyrosine synthesis, neurotransmitter synthesis, and functioning as a reducing antioxidant agent by donating electrons and hydrogen ions. Self-reported dietary antioxidant capacity data from 7642 participants in the NHANES study (2007–2018) showed a significant reduction in the risk of all-cause mortality among patients with early-stage CKM [47,48]. Applying the oxidative balance score (OBS), a marker of inflammation, to machine-learning algorithms, including XGBoost, Liu and collaborators showed that OBS was negatively correlated with CKM in data obtained from NHANES cohorts (1999 to 2018), suggesting a protective anti-inflammatory effect in CDK syndrome [49]. Comparable results were observed in a cross-sectional study using the Composite Dietary Antioxidant Index (CDAI) on NHANES data (2007–2018), which found that a higher CDAI was associated with lower odds of developing CKM syndrome, suggesting that a diet rich in antioxidants could help reduce the risk of CKM [50,51].

Similarly, Duan and colleagues analyzed data from 7643 participants in the NHANES cohort (2007–2018) with CKM Stages 0–3. They reported that dietary carotenoids and fat-soluble vitamin E levels, as a proxy for dietary antioxidant capacity, were associated with reduced all-cause mortality in patients with CKM [47]. This observation suggests that multi-dietary antioxidant intake may target the oxidative stress pathway and thus may be helpful in the prevention and management of patients with CKM. Similar findings were reported in another study using data from 11,073 participants in the same NHANES cohort (2007–2018), which included patients with advanced CKM (Stages 3–4), and found that higher CDAI scores were negatively correlated with CKM stage [50]. Likewise, data from a different NHANES cohort (2001–2006) of participants with CKM syndrome Stages 3–4 (n = 1285) found that higher serum carotenoid concentrations were associated with lower all-cause mortality in US adults with advanced CKM syndrome [52]. Along the same line, Chen and colleagues also found a negative correlation between fasting serum carotenoid levels (α-carotene, β-carotene, α-cryptoxanthin, lutein/zeaxanthin, and lycopene) and advanced CKM in the NHANES participants cohort 2017–2018 (n = 1671) [53]. Taken together, the observational studies suggest a correlation between water-soluble and fat-soluble vitamins and protection from CKM.

However, it is worth noting that observational studies are correlational, not causal, and the main limitation of the dietary intake data obtained from NHANES is that it is self-reported and subjective; therefore, it is not quantifiable. Since observational studies provide evidence of correlation between vitamin intake and CKM, it remains to be seen whether randomized controlled trials and causal inference studies would reveal a causal relationship between fat and water-soluble vitamin intake and protection against, or a reduction in, comorbidities associated with CKM.

### 5.5. CKM and Minerals

Although minerals do not provide energy, many minerals serve as cofactors for metalloenzymes and are required for energy-yielding biochemical reactions. In addition, dietary intake of minerals is essential for cellular functions and electrolyte balance. Hu and colleagues studied the dietary intake of several major minerals, including calcium; phosphorus; potassium; magnesium; trace minerals, such as iron, copper, and zinc; and ultra-trace minerals, such as selenium, individually and in mixtures, using data from 15,233 participants in NHANES (2003–2018) in US adults aged 20–79 years [54]. The authors did not find an association between individual or mixture intakes of these minerals, except for copper intake. The data showed that higher copper intake (>0.8 mg/day) was associated with a reduced risk of advanced CKD compared with lower intake (<0.8 mg/day) in US adults [54].

Electrolytes such as sodium, chloride, calcium, phosphorus, potassium, and magnesium play an integral role in maintaining water and electrolyte balance. Elevated potassium level (>5 mEq/L), known as hyperkalemia, is associated with increased risk of cardiac toxicity and muscle weakness [55]. In contrast, higher magnesium depletion scores have been reported to be associated with increased odds of advanced CKM in the NHANES 1999–2018 cohort, which included 18,038 US adult participants aged 20–79 years [56]. In a respective cohort study, data extracted from the electronic medical records of 270 patients admitted with acute myocardial infarction (MI) associated with CKM revealed that in-hospital mortality in patients with low serum calcium (0–2.10 mmol/L) was significantly higher than mortality in patients with higher serum calcium levels (>2.11 mmol/L), suggesting that serum calcium could be used a predictor of mortality in patients with MI and CKM, particularly in older adults (≥76 years) [57]. A comprehensive, multidisciplinary approach to managing electrolyte levels has been recommended by the international multidisciplinary consensus panel on emergency care for patients with CKM [55].

Finally, heavy metals such as cadmium, lead, cobalt, and barium are not catabolized in the body, and upon exposure, they can accumulate in major organs, leading to tissue and organ damage, with detrimental effects on health. Liu and colleagues analyzed data from 5221 participants in the NHANES cohort, 2005–2018. They found that an elevated urinary heavy metal mixture composed of antimony, barium, cadmium, cesium, cobalt, lead, molybdenum, thallium, and tungsten was associated with advanced-stage CKM, with the strongest correlation observed for cobalt levels in this cohort [58,59]. Additionally, He and colleagues analyzed data from 5865 participants in the NHANES (2011–2016) cohort, which revealed that higher serum cadmium levels were associated with increased progression of CKM (Odds Ratio (OR) = 1.21; Confidence Interval (CI) 1.05–1.40) and all-cause mortality in advanced-stage CKM [60]. Similarly, using an exposome-wide approach, Ma and colleagues reported that exposure to heavy metals, including tin, volatile organic compound metabolites, and polycyclic aromatic hydrocarbons (PAHs), was associated with advanced CKM [61]. As a complementary approach, using NHANES data from the 1999–2018 cohort (n = 6650 participants), Zhang and colleagues reported a positive correlation between urinary barium, cadmium, cobalt, cesium, molybdenum, antimony, thallium, and tungsten, both individually and in mixtures, across different stages of CKM [62]. The authors conducted a mediation analysis, which revealed that this association could be due in part to oxidative stress, inflammation, and biological aging [62]. To further explore the effects of environmental metal exposure on CKM syndrome, Zhang et al. [63] analyzed data from 1816 participants of the NHANES (2011–2016) cohort using the weighted quantile sum regression and reported that mixed metal exposure was associated with increased risk of CKM (effect size β = 0.502; *p* = 0.013). The author further suggested that cobalt is a key driver of the metabolic and immune dysregulations as identified by the Adverse Outcome Pathway framework [63].

### 5.6. CKM and Different Diets

Several investigators have used the US national NHANES data to correlate different diets and dietary patterns to the risk of CKM. However, observational studies can only establish correlations, not causation. Shao et al., using the NHANES data (2005–2018) cohort, which included 16589 adult participants (30 years and older), reported that higher-quality dietary pattern measured by the alternative Mediterranean diet score was associated with a lower risk of all-cause mortality among people with CKM syndrome [64].

#### 5.6.1. CKM and the Dietary Approaches to Stop Hypertension (DASH) Diet

Akubo and colleagues used the DASH adherence score, based on higher intake of fruits, vegetables, and whole grains; and reduced intake of salt and sugar-sweetened beverages and processed/red meat. They reported an inverse correlation between high DASH adherence and lower risk of CKM [65]. The findings support the inclusion of the DASH diet as a nutrition intervention for the prevention and management of CKM syndrome [65]. On the other hand, Baker-Smith and colleagues did not find an association between the DASH diet and DKM Stages 1 and 2 in an adolescent-representative sample from a cross-sectional analysis of NHANES data (2017–2020 cohort) [66]. Thus, further research is warranted to determine the impact of the DASH diet on the management of CKM syndrome.

#### 5.6.2. CKM and Plant-Based Diet

A plant-based diet is an eating pattern that focuses on vegetable/plant-based oils and consuming fruit, vegetables, grains, legumes, nuts, and seeds. Studies have shown that a plant-based diet decreases diabetic dyslipidemia, hypercholesterolemia, and/or hypertriglyceridemia [67,68,69]. Therefore, we reviewed the available observational studies focusing on plant-based diets and CKM syndrome.

Using NHANES longitudinal data (1999–2002) cohort, Xiao et al. showed that a plant-based diet was associated with reduced all-cause mortality and cardiac mortality in participants with CKM syndrome [70]. It is important to note that the dietary data are from a self-reported 24 h dietary recall and have been converted to food-pattern equivalents. The authors classified the food pattern as six healthy plant foods, five unhealthy plant foods, and six animal-based foods. It is unclear, however, why the authors classified fruit as healthy but fruit juice as unhealthy. It is worth noting that studies reported to date have been observational studies, mainly using the NHANES database, and no randomized clinical trials have been reported.

#### 5.6.3. CKM and Anti-Inflammatory Diet Pattern

There are overlapping characteristics between a plant-based diet and an anti-inflammatory diet. However, in the anti-inflammatory diet, the researchers address the anti-inflammatory properties of food and report on the anti-inflammatory or pro-inflammatory cytokines, such as interleukins, tumor necrosis factor, and other cytokines, as biomarkers, and calculate the DII [71]. Shang and colleagues found that the CKM risk and progression were correlated with an inflammatory diet pattern using NHANES (2005–2018) cohort data and the 24 h self-reported dietary recall. [72]. The investigators used the DII, which includes 45 pro- and anti-inflammatory food parameters, scored based on whether they increase or decrease levels of inflammatory cytokines, including IL-1β, IL-4, IL-6, IL-10, TNF-α, and C-reactive protein [73]. Other investigators also reported the association between the DII and the progression of CKM using NHANES data [74,75]. Similarly, studies applying the Dietary Index for Gut Microbiota (DI-GM), which assesses the effects of diet on the gut microbiome, using NHANES cohort data (2007–2016), showed that participants with higher DI-GM (>6) had a lower CKM risk compared to those with DI-GM <3 (OR = 0.77, 0.59–0.99; *p* < 0.05) [76]. Comparable findings from NHANES data (2007–2018) showed that low DI-GM was associated with an increased prevalence of CKM, and vice versa [77,78]. Similarly, Yan et al. reported that the DI-GM score was inversely correlated with advanced CKM in a cross-sectional study of 12,068 participants from the NHANES cohort (2007–2020) [78].

#### 5.6.4. CKM and Lifestyle Intervention, Physical Activity, and Health Behavior

Using NHANES (2007–2018) cross-sectional data from 9447 adults, Chen and colleagues reported that physical inactivity, smoking, and poor diet quality were positively associated with higher CKM risk categories [79]. Similar findings were reported from other investigators using NHANES data from multiple cohorts [80,81,82,83].

Conversely, participants with a higher lifestyle score were less likely to have advanced-stage CKM [80]. Similarly, in a study of 147 patients with CKD, consumption of a healthy plant-based diet, assessed using a food frequency questionnaire developed for the Asian population, was associated with a lower risk of CKM syndrome [84]. In a 10-year prospective cohort study that enrolled 100,727 participants in the China CVD and Cancer Cohort, Li and colleagues reported that CKM stages were associated with increased CVD events, with CKM Stage 4 having an Hazard Ratio (HR) of 5.95 (4.74–7.45) compared to CKM Stage 0 [85]. Furthermore, participants with Stage 1 or 2 CKM and an optimal cardiovascular health matrix of the Life Essential Health 8 (LE 8), including diet, physical activity, sleep, BMI, blood lipids, low nicotine exposure, blood pressure, and blood glucose, did not show an increased risk of CVD adverse events, suggesting that improving the LE8 would be beneficial in health improvement and decreasing CVD events, including myocardial infarction, stroke, and cardiac-related mortality [85]. Taken together, the findings suggest that a healthier lifestyle and higher intake of plant-based foods were associated with better staging and better health outcomes in patients with CKM. However, causal-inference studies and randomized controlled trials are warranted to establish causality.

## 6. CKM and Liver Diseases

Although the CKM framework does not include the liver, it is reasonable to consider liver diseases when addressing CKM syndrome, given that the liver is a central metabolic hub. The liver plays an integral role in carbohydrate, protein, and lipid metabolism; nutrient distribution; drug detoxification; bile and protein synthesis; immune functions; and antibody production. Given its role in metabolic homeostasis, lipoprotein synthesis for lipid transport, chemical detoxification, and the interconnectivity of integrated networks and biological systems, we hypothesized that the liver may contribute to the development and progression of CKM. Indeed, other investigators have suggested a role for liver diseases such as metabolic dysfunction-associated steatohepatitis (MASH) and metabolic dysfunction-associated steatotic liver disease (MASLD) in CKM staging and risk stratification [86,87,88].

Recent studies provide evidence for the liver’s contribution to the concept of CKM syndrome [88,89,90,91,92,93,94,95,96,97,98,99,100]. As such, Hu and colleagues enrolled 6186 participants in a community-based cross-sectional study in China and reported that MASLD and MASLD-related liver fibrosis are associated with advanced CKM syndrome in this population, with findings similar to those from the NHANES data in the US [88]. Additionally, Ke et al. reported that the estimated glucose disposal rate, as a proxy for IR, could help stage CKM based on studies using NHANES data from 1999 to 2018 [89]. Along the same line, Kipp and colleagues utilized the gut microbiome’s conversion of bilirubin to urobilirubin in the gut–liver–kidney axis as a biomarker in CKM syndrome [90]. Similarly, Gan et al. reported that a 10-protein score for fat liver involved in lipid and carbohydrate metabolism may predict CKM risk [99]. 

## 7. Glucagon-like Peptide-1 (GLP-1) and CKM

GLP-1 was originally prescribed in the treatment of diabetes mellitus because it reduces glucagon, the insulin antagonist, and stimulates the pancreas to release insulin when blood glucose is elevated. In addition, it reduces appetite and slows gastric emptying, thereby reducing body weight and obesity [101]. But recently, it has been shown to reduce albuminuria and slow CDK progression, particularly for people with diabetes mellitus [101,102,103]. Additionally, it has been reported to lower blood pressure, improve cholesterol levels, and thereby reduce CVD risk [104]. Furthermore, GLP-1 also reduced inflammation in MASLD, thereby decreasing liver dysfunction and failure [105]. Taken together, the evidence suggests that the GLP-1 therapy could benefit patients with CKM.

## 8. Summary and Conclusions

Integrated body systems contribute to the development and progression of CKM. The risk of mortality increases when comorbidities related to CKD and CVD are present, suggesting that these clinical conditions and age differentials could be drivers for increased mortality at Stages 3 and 4 of CKM syndrome. As adipose dysfunction, excess adiposity, inflammation, and abdominal obesity are associated with the early stages of CKM syndrome, it is reasonable to propose that precision nutrition interventions and healthier lifestyle changes could be actionable strategies to reduce insulin resistance, obesity, and pre-diabetes mellitus, thereby either preventing the development of CKM syndrome or reducing the progression to advanced stages. Further research is warranted to document the impact of precision nutrition, dietary patterns, and lifestyle-centered interventions on the prevention, early detection, and management of CKM syndrome. The current evidence is mainly derived from observational studies, which show only correlations, but provide a rationale for conducting experimental, causal inference studies, and clinical trials to assess the possible role of personalized and precision nutrition in the prevention, early detection, and interventions for CKM. These questions remain unanswered and warrant future research.

**Figure 1:**
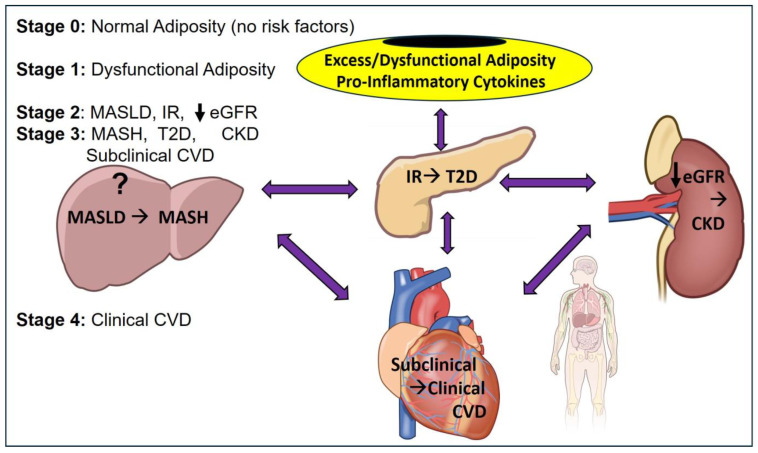
CKM Syndrome Hypothesized Cross-Talk and Integrated Systems Pathophysiology. The question mark on the liver image indicates the hypothetical theory involvement of the liver as part of the disease.

Future studies should examine the effects of dietary interventions, including the Mediterranean diet, ketogenic diet, intermittent fasting, and very low calorie diet on the management of CKM syndrome. Based on the current evidence and consistent with the framework proposed by Theodorakis and colleagues [95,106], we hypothesize that tissue crosstalk and system integration among cardiovascular, hepatic, renal, and metabolically active adipocytes drive the progression of CKM syndrome (Figure 1). It would be worth considering including MASLD and MASH within the CKM framework. Accordingly, we propose a CLKM framework (Cardiovascular–Liver–Kidney–Metabolic Syndrome) or CRHM (Cardiovascular–Renal–Hepatic-Metabolic Syndrome) to develop strategies for prevention, early detection, monitoring, and therapeutic interventions.

## Data Availability

This manuscript is a narrative review of peer-reviewed published work. No new data were created in this study.

## Abbreviations

The following abbreviations are used in this manuscript:

AHA	American Heart Association
AGE	Antiglycation products
BMI	Body mass index
BRI	Body rounded index
CDAI	Composite Dietary Antioxidant Index
CDK	Chronic kidney disease
CETP	Cholesteryl ester transfer protein
CHARLS	China Health and Retirement Longitudinal Study
CI	Confidence interval
CKM	CKM Syndrome
CLKM	Cardiovascular–Liver–Kidney–Metabolic Syndrome
CVD	Cardiovascular disease
DASH	Dietary approaches to stop arterial hypertension
DEXA	Dual X-ray absorptiometry
DI-GM	Dietary index for gut microbiota
DII	Dietary Inflammatory Index
eGRF	Estimated glomerular filtration rate
FLX	Dietary additive supplement
GLP-1	Glucagon-Like Peptide 1
IL-10	Interleukin-10
HDL	High Density Lipoprotein
HR	Hazard Ratio
IL-1β	Interleukin-1 βeta
IL-4	Interleukin-4
IL-6	Interleukin 6
IR	Insulin resistance
KDIGO	Kidney disease improving global outcomes.
LE8	Life Essential 8
LDL	Low Density Lipoprotein
MASH	Metabolic dysfunctional-associated steatohepatitis
MASLD	Metabolic dysfunction-associated steatotic liver disease
Mets	Metabolic syndrome
MI	Myocardial infarction
NHANES	National Health and Nutrition Examination Surveys
NrF2	Nuclear factor erythroid 2-related factor 2
OBS	Oxidative balance score
OR	Odds ratio
PAH	Polycyclic aromatic hydrocarbon
RCT	Randomized controlled trial
TNFα	Tumor necrosis factor alpha
TyG	Triglyceride glucose index
TCA	Tricarboxylic acid cycle
T2D	Type 2 diabetes mellitus
WC	Waist circumference
